# Tolcapone Potently Inhibits Seminal Amyloid Fibrils Formation and Blocks Entry of Ebola Pseudoviruses

**DOI:** 10.3389/fmicb.2020.00504

**Published:** 2020-04-30

**Authors:** Mengjie Qiu, Zhaofeng Li, Yuliu Chen, Jiayin Guo, Wei Xu, Tao Qi, Yurong Qiu, Jianxin Pang, Lin Li, Shuwen Liu, Suiyi Tan

**Affiliations:** ^1^Guangdong Provincial Key Laboratory of New Drug Screening, School of Pharmaceutical Sciences, Southern Medical University, Guangzhou, China; ^2^Laboratory Medicine Center, Nanfang Hospital, Southern Medical University, Guangzhou, China; ^3^School of Pharmacy, Guangdong Medical University, Dongguan, China

**Keywords:** Ebola virus, tolcapone, semen-derived enhancer of viral infection (SEVI), sexual transmission, entry inhibitor

## Abstract

Ebola virus (EBOV), the causative pathogen of the deadly EBOV disease (EVD), can be transmitted *via* sexual transmission. Seminal amyloid fibrils have been found enhancers of EBOV infection. Currently, limited preventive vaccine or therapeutic is available to block EBOV infection through sexual intercourse. In this study, we repurpose tolcapone, a US Food and Drug Administration (FDA)-approved agent for Parkinson’s disease, as a potent inhibitor of seminal amyloid fibrils, among which semen-derived enhancer of viral infection (SEVI) is the best-characterized. Tolcapone binds to the amyloidogenic region of the SEVI precursor peptide (PAP248–286) and inhibits PAP248–286 aggregation by disrupting PAP248–286 oligomerization. In addition, tolcapone interacts with preformed SEVI fibrils and influences the activity of SEVI in promoting infection of pseudovirus (PsV) carrying the envelope glycoprotein (GP) of the EBOV Zaire or Sudan species (Zaire PsV and Sudan PsV, respectively). Tolcapone significantly antagonizes SEVI-mediated enhancement of both Zaire PsV and Sudan PsV binding to and subsequent internalization in HeLa cells. Of note, tolcapone is also effective in inhibiting the entry of both Zaire PsV and Sudan PsV. Tolcapone inhibits viral entry possibly through binding with critical residues in EBOV GP. Moreover, the combination of tolcapone with two small-molecule entry inhibitors, including bepridil and sertraline, exhibited synergistic anti-EBOV effects in semen. Collectively, as a bifunctional agent targeting the viral infection-enhancing amyloid and the virus itself during sexual intercourse, tolcapone can act as either a prophylactic topical agent to prevent the sexual transmission of EBOV or a therapeutic to treat EBOV infection.

## Introduction

Ebola virus (EBOV) is the causative pathogen of the deadly EBOV disease (EVD). It belongs to the *Filoviridae* family and can be classified into six distinct species, including *Zaire ebolavirus* (ZEBOV), *Sudan ebolavirus* (SUDV), *Tai Forest ebolavirus* (TAFV), *Bundibugyo ebolavirus* (BDBV), *Reston ebolavirus* (RESTV), and the newly identified *Bombali ebolavirus* (BOMV) (Van Kerkhove et al., 2015; Goldstein et al., 2018). Among them, ZEBOV have the highest case-fatality rates (60–90%) followed by those for the SUDV (40–60%). Other EBOVs have been associated with rates of mortality of 0–25% (Weyer et al., 2015; Thorson et al., 2016). After the first recognized outbreak in 1976, numerous EBOV outbreaks have occurred over the years. However, it was only until the recent outbreak of EBOV in 2014–2016, which caused approximately 28,200 cases and 11,300 deaths, highlighted the danger and global impact of this pathogen. Although the epidemic has now subsided, the increase in outbreak frequency, number of cases, and associated social and economic cost necessitates the need for effective vaccines and drugs to combat this pandemic threat (Dhama et al., 2018).

EBOVs are usually transmitted through direct contact with EVD patients, whose body fluids contain high titers of viruses (Lanini et al., 2015). The shedding of infectious virus and virus genome in semen has been documented since 1976 (Rodriguez et al., 1999; Bausch et al., 2007; Mate et al., 2015; Barnes et al., 2017). However, sexual transmission of EBOV was only recently confirmed during the 2014–2016 outbreaks in West Africa (Mate et al., 2015; Keita et al., 2016; Thorson et al., 2016). Several follow-up studies have shown that EBOV could persist in semen up to 2 years after the onset of disease (Barnes et al., 2017; Deen et al., 2017; Keita et al., 2017), which raises a critical concern for the increasing risk of sexual transmission of EBOVs by asymptomatic survivors (Fischer et al., 2017). Although WHO recommends safe sexual practices, including abstinence or condom use, for at least 1 year after the onset of symptoms (Thorson et al., 2016; Keita et al., 2017), it might be too short based on the fact of long-term persistence of virus genome in semen (Fischer et al., 2017) and not practicable because 74% of male EVD survivors were reported to not prefer to use condoms during sexual intercourse. Therefore, an effective and safe microbicide, administered vaginally by women without the need for approval and cooperation from sexual partners in the low-income countries to prevent EBOV sexual transmission before the next outbreak, is urgently needed.

Human semen contains amyloid fibrils that could greatly enhance infection of pathogens of sexually transmitted infections (STIs), including HIV-1, herpes simplex virus (HSV), cytomegalovirus (CMV) (Tang et al., 2013; Torres et al., 2015), etc. Among them, the well-documented pathogen is HIV-1 (Münch et al., 2007; Roan et al., 2009, 2011; Kim et al., 2010). Seminal amyloid fibrils are highly cationic and are made up of naturally occurring peptide fragments, including prostatic acid phosphatase (PAP248–286 and PAP85–120) and the homologous proteins semenogelin 1 (SEM1) and semenogelin 2 (SEM2) (Arnold et al., 2012). Semen-derived enhancer of viral infection (SEVI), formed by PAP248–286 self-aggregating, is the best-characterized seminal amyloid (Lee and Ramamoorthy, 2018). Recently, infection by EBOV with sexual transmission routes has been found to also be enhanced by SEVI (Bart et al., 2018). These seminal fibrils act in a conformation- and charge-dependent manner to increase infection by promoting viral entry into target cells, which is similar with that reported for HIV-1 (Roan et al., 2009; Bart et al., 2018). SEVI specifically enhances EBOV binding to and subsequent internalization and macropinocytotic uptake into target cells (Bart et al., 2018). More importantly, seminal amyloid fibrils establish a microenvironment that might be beneficial for EBOV persistence (Bart et al., 2018). Of note, the potency of antiretroviral agents decreases in the presence of seminal amyloids/semen (Zirafi et al., 2014). Therefore, seminal amyloid fibril serves as a novel drug target for sexually transmitted EBOV. Although there are five compassionate-use investigational therapeutics for treatment of EVD, including the antivirals favipiravir (Sissoko et al., 2016) and GS-5734 (Warren et al., 2016) and antibody therapeutics mAb114 (Corti et al., 2016), Zmapp (Qiu et al., 2014), and REGN3470-3471-3479 (Sivapalasingam et al., 2018), none of these are US Food and Drug Administration (FDA)-approved or have been tested effective in the context of EBOV sexual transmission. Seeking a time-saving, cost-saving, and multifunctional candidate microbicide is of particular importance.

During our review of the amyloid inhibitors in the literature in our previous study (Lump et al., 2015; Xun et al., 2015; Ren et al., 2018; Zhang et al., 2018; Li et al., 2019; Tan et al., 2019), we noticed that tolcapone, an active catechol-*O*-methyltransferase (COMT) inhibitor clinically used as an adjunct to levodopa/carbidopa for Parkinson’s disease (Olanow and Watkins, 2007; Müller, 2015), possessed inhibitory activity against transthyretin (TTR) amyloidosis, which is a plasma homotetrameric protein associated with fatal systemic amyloidoses. Tolcapone was also shown to be effective in treating amyloid transthyretin (ATTR) amyloidosis *in vivo* (Gamez et al., 2019). It shows high binding affinity to the thyroxine binding sites of the native tetrameric form of TTR, preventing its dissociation into monomers. The drug also exhibits fibril disruption activity *in vitro* (Sant’Anna et al., 2016). More importantly, tolcapone displays inhibitory activity against EBOV infection by targeting the interaction between viral protein 35 (VP35) and nucleoprotein (NP) at the late stage of viral infection (Liu et al., 2017). Therefore, we questioned whether tolcapone could be a repositioned compound with simultaneously inhibitory effects on seminal amyloidogenic peptide fibrillogenesis and EBOV infection to stop the sexual transmission of EBOV.

In the current study, we sought to elucidate the inhibitory effects of tolcapone on the seminal amyloid fibrils and the underlying mode of action. We also reported for the first time that tolcapone was a potent EBOV entry inhibitor. It exhibited a synergistic effect in combination with other entry inhibitor-based antiviral agents. As well, the safety profile of tolcapone *in vitro* was explored.

## Materials and Methods

### Reagents

Congo red, ammonium persulfate, DL-dithiothreitol (DTT), tris (2, 2-bipyridyl) dichlororuthenium (II) [Ru (bpy)_3_^2^^+^], 3-(4,5-dimethylthiazol-2-yl)-2,5-diphenyltetrazolium bromide (MTT), polyethyleneimine (PEI), Triton, thioflavin T (ThT), and SYPRO Orange Protein dye were purchased from Sigma (St. Louis, MO, United States). Chemicals, including tolcapone, bepridil hydrochloride (bepridil), sertraline hydrochloride (sertraline), and favipiravir, were bought from TargetMol (United States). PAP248-286, PAP248-286(Ala), and SEM1 86-107 (>95% purity) were synthesized by Scilight-Peptide (Beijing, China). Peptides PAP 248–286 and SEM1 86–107 were first dissolved in phosphate buffered saline (PBS) at the concentration of 1 mM, and fibril formation was promoted by agitation at 37°C for 24–72 h at 1,400 rpm by using an Eppendorf Thermomixer. Semen (SE) samples were obtained from healthy lab members with written informed consent in accordance with the Declaration of Helsinki, and the study was approved by the Human Ethics Committee of Southern Medical University, China. Ejaculates were liquefied as soon as collected for 30 min at room temperature. Seminal fluid (SE-F), representing the cell-free supernatant of SE, was collected by centrifugation of 1 ml SE for 15 min at 10,000 rpm and stored in 1-ml aliquots at −20°C. HEK-293T cells, Huh-7 cells, and HeLa cells were grown in Dulbecco’s modified Eagle’s medium (DMEM) supplemented with 10% fetal bovine serum (FBS; ExCell Bio) and 1% penicillin/streptomycin at 37°C and 5% carbon dioxide (CO_2_). A549 cells and THP-1 cells were maintained in RPMI 1640 medium supplemented with 10% FBS and 1% penicillin/streptomycin at 37°C and 5% CO_2_. Human monocyte-derived THP-1 cells were differentiated into macrophages by using phorbol-12-myristate-13-acetate (PMA; Sigma) before they were used as target cells (Johnson et al., 2014). Plasmids of HIV-1HXB2 (X4 strain), HIV-1 JR-FL (R5 strain), vesicular stomatitis virus-G (VSV-G), pNL4-3.Luc.R^–^E^–^, and anti-p24 monoclonal antibody (183-12H-5C) were obtained from the National Institutes of Health AIDS Research and Reference Reagent Program. Plasmids of glycoproteins (GPs) of ZEBOV and SUDV were prepared as described elsewhere (Li et al., 2017). The soluble Zaire GP [without the mucin-like domain (MLD) and the transmembrane (TM) domain] that possesses the native trimer conformation of GP is a gift from Dr. Lu Lu of Fudan University, China.

### Congo Red Staining Assay

Peptide PAP248–286 or PAP248–286(Ala) at 220 μM in PBS was mixed with inhibitor at various concentrations and then agitated at 1,400 rpm at 37°C by using an Eppendorf Thermomixer. To monitor the reaction kinetics, 10-μl aliquot of the reaction mixture were withdrawn from each tube at different time points and detected by a Congo red kit as previously described (Tan et al., 2019). The Congo red staining measurements were plotted as a function of time and fitted by a sigmoidal curve using Origin data analysis software to determine lag time (*t*_*lag*_) (Nakka et al., 2018).

### Thioflavin T Fluorescence Assay

Ten microliters of sample prepared as described above was mixed with 190 μl of thioflavin T (ThT) working solution (50 μM) (Nilsson, 2004). The fluorescence of the mixture was measured using an RF-5301 PC spectrofluorophotometer (Shimadzu) at an excitation wavelength of 440 nm and an emission of 485 nm.

### Transmission Electron Microscopy Analysis

Fibrils were generated as described in the Congo red staining section. At indicated time points, aliquots were removed from respective reactions and subjected to transmission electron microscopy (TEM) analysis (Tan et al., 2013). The morphology of SEVI or seminal fibrils in the presence and absence of tolcapone (the final concentration of PAP248–286 was 66 μM, and the final dilution of SE-F was 1:5) was visualized using an H-7650 TEM (Hitachi Limited, Tokyo, Japan).

### Circular Dichroism Spectroscopy

PAP248–286 at 220 μM was incubated with or without tolcapone at various concentrations and agitated at 1,400 rpm at 37°C, after which circular dichroism (CD) measurements were performed using a J-715 spectrometer (Jasco, Japan). The CD spectra were recorded in the 190–280-nm region at a scan speed of 50 nm⋅min^−1^, with a bandwidth of 1 nm and a time constant of 2 s. All measurements were done at room temperature, and the final concentration of each sample was 66 μM. The baseline was corrected to values for the buffer content with different concentrations of tolcapone. The CD curves were smoothed using GraphPad Prism software.

### Tricine-Sodium Dodecyl Sulfate-Polyacrylamide Gel Electrophoresis

PAP248–286 was incubated with tolcapone at indicated concentrations at 37°C for 30 min. Mixtures were then centrifuged at 5,000 rpm for 5 min. The free PAP248–286 in the supernatant was mixed with loading buffer and boiled at 100°C for 10 min. Then, the samples were separated by 16% gradient tricine-sodium dodecyl sulfate-polyacrylamide gel electrophoresis (SDS-PAGE). Peptides were visualized by Coomassie blue staining as indicated elsewhere (Tan et al., 2013).

### Photo-Induced Cross-Linking of Unmodified Proteins

Samples were chemically cross-linked using photo-induced cross-linking of unmodified proteins (PICUPs) as previously reported (Ren et al., 2018). Firstly, PAP248–286 monomer at 220 μM was incubated with various concentrations of tolcapone or suramin (15, 20, and 30 μM) at 37°C for 30 min. Next, 1 μl of 40 mM tris (2,2-bipyridyl)dichlororuthenium (II) [Ru (bpy) 32+] and 1 μl of 800 mM ammonium persulfate were added to 18 μl of the mixed sample. The mixture was then exposed to visible light for 8 s, and the cross-linking reaction was terminated by the addition of 2 μl of 5 M DTT. Non-cross-linked PAP248–286 monomer served as a negative control. The samples were separated by 16% gradient tricine-SDS-PAGE, and Coomassie blue staining was used to determine the frequency distribution of monomers and oligomers of PAP248–286.

### Preparation of Pseudoviruses and Viral Infection Assay

Pseudoviruses were prepared as previously described (Chan et al., 2000; Xun et al., 2015; Li et al., 2017; Qiu et al., 2019). Briefly, HEK-293T cells were co-transfected with a pNL4-3.Luc.R^–^E^–^ plasmid and different EBOVs Env-encoding plasmids derived from Zaire pseudovirus (Zaire PsV), Sudan pseudovirus (Sudan PsV), HIV-1 Env-encoding plasmids derived from HIV-1HXB2 (X4 strain), HIV-1JR-FL (R5 strain), and VSV-G Env-encoding plasmids by using a PEI transfection reagent. Cell culture supernatants containing pseudoviruses were collected 72 h after transfection and centrifuged at 3,500 rpm for 15 min to remove detached cells and cell debris and then stored in a -80°C refrigerator. The pseudoviruses were quantitated by determination of the level of p24 antigen by ELISA as described elsewhere (Li et al., 2010). In detail, 5 μg/ml of 183-12H-5C diluted in sodium carbonate buffer (0.05 M, pH = 9.6) was coated onto all wells of a 96-well polystyrene plate (Corning, United States) at 4°C overnight. After blocking with 2% skimmed milk powder, the lyzed pseudovirus serially diluted in PBS was added to the wells, and the plate was incubated at 37°C for 1 h. Rabbit polyclonal to HIV-1 p24 antibody (Abcam, United Kingdom) at a 1:2,000 dilution was added into the wells, and the plate was incubated at 37°C for 1 h and then further incubated with peroxidase affinipure goat anti-rabbit IgG (H+L) (Abcam, United Kingdom) at a 1:3,000 dilution ratio for one more hour at 37°C. The absorbance of the wells was measured after reacting with tetramethylbenzidine (Sigma, United States). When stored at −80°C in 6 months, the luciferase values in HeLa, A549, THP-1, and Huh-7 cells showed no significant difference compared with that at 0 months, indicating that the activity of pseudovirions was relatively stable at −80°C for 6 months.

PAP248–286 or whole SE-F was agitated to allow fibril formation with or without tolcapone as described in the Congo red staining section. Samples at indicated time points were collected and centrifuged to pellet the fibrils at 12,000 rpm for 5 min to remove the remaining free tolcapone. The pellets were resuspended in culture medium and used to determine their abilities to enhance Ebola pseudoviruses infection as described previously (Xun et al., 2015). Briefly, the pellets were mixed with Ebola pseudovirus (10 ng p24) for 10 min at room temperature. The pseudovirus–fibril mixtures were then used to infect various target cells (10^4^/well) that have been cultured overnight. Luciferase activity in triplicate well was measured 72 h postinfection using luciferase assay kit (Promega) according to the manufacturer’s instruction. The enhancement of viral infection was shown relative to those measured in the absence of peptide (Tan et al., 2019). The final concentration of PAP248–286 and SE-F in the infection assay was 11 μM and 5%, respectively.

To confirm that tolcapone could diminish the mature fibril-mediated enhancement of viral infection, SEVI or whole SE-F, which has been agitated for 8 h, was incubated with graded concentration of tolcapone at 37°C for 15 min. To eliminate the anti-EBOV activity of tolcapone itself as much as possible, the SEVI or SE-F and tolcapone mixtures were centrifuged at 12,000 rpm for 5 min to discard the free tolcapone. Then, the pellets were dissolved in fresh medium and were tested on their abilities to enhance EBOV infection as described above. SEVI was tested at a final concentration of 11 μM, and SE-F was used at a final concentration of 5%.

### Measurement of the Entry Inhibition Activity of Tolcapone Individually and in Combination With Entry-Inhibitory Antivirals in Semen

A pseudo-EBOV inhibition assay was performed in HeLa, A549, THP-1, and Huh-7 cells as previously described (Li et al., 2017). Briefly, cells were seeded at 10^4^ cells/well into a 96-well plate and incubated overnight at 37°C. EBOV PsV (60 ng p24) was incubated with a serially diluted inhibitor for 30 min at 37°C, followed by the addition of cultured cells. The cells were incubated with or without pseudovirus as virus control and cell control, respectively. The culture was replaced with fresh medium 14 h post-infection, and luciferase activity was detected 72 h later. The effective 50% inhibitory concentration (IC_50_) was calculated using the Compusyn software (Xu et al., 2014).

For the inhibitor combination assay, tolcapone was mixed with entry inhibitor at the indicated molar concentration ratio, while tolcapone and each entry inhibitor were included as controls. The mixtures were serially diluted, incubated with SE-F, and tested for their inhibitory activities on Zaire PsV infection in HeLa cells as described above. SE-F was used at a final dilution of 1:20 to avoid cytotoxicity. Each sample was tested in triplicate, and data were analyzed for synergistic effect by calculating the combination index (CI), using the CalcuSyn program. CI values of <1 and >1 indicate synergy and antagonism, respectively, and synergy was divided into different strengths, according to CI values, as follows: <0.1 indicates very strong synergism; 0.1–0.3 indicates strong synergism; 0.3–0.7 indicates synergism; 0.7–0.85 indicates moderate synergism; and 0.85–0.90 indicates slight synergism (Qi et al., 2017). Fold of potency enhancement was calculated with the ratio of concentrations of inhibitor tested alone and in combination.

### Binding/Internalization Assays

SEVI was incubated with or without tolcapone at 37°C for 15 min, and the pellets were collected by centrifugation at 12,000 rpm for 5 min to remove free tolcapone. Afterward, the fibril pellets were resuspended and further incubated with Zaire-PsV (30 ng p24) at 37°C for 15 min and then added to HeLa cells on ice and either lysed in 1% Triton after 1 h (binding) or warmed to 37°C for an additional 1 h to allow internalization, then washed, trypsinized, and lysed (internalization) (Bart et al., 2018). The amounts of binding or internalized virus were determined by Western blotting.

### Mass Spectrometric Analysis

Mass spectrometric detection was performed on an AB Sciex API4000 Triple Quad rupole mass spectrometer running in positive ionization mode. Electrospray (ESI) voltage was set at 5.5 kV, source temperature at 0°C. Ion source gas 1 (desolvation gas-nitrogen) pressure was set at 15 psig, ion source gas 2 (nebulizer gas-nitrogen) at 10 psig, and curtain gas flow at 10 psig. Electron multiplier CEM was set at 2,000 V, and entrance potential was fixed at 10 V. The final concentration of PAP248–286 is 2 μg/ml, and tolcapone is 20 μg/ml. The mixture of PAP248–286 and tolcapone above was agitated at 1,400 rpm at 37°C in an Eppendorf Thermomixer before being detected by mass spectrometry.

### Zeta Potential Measurement

SEVI at 220 μM was treated with tolcapone at indicated concentrations. Mixtures were centrifuged for 10 min at 10,000 rpm. The pellets were resuspended in 1 ml of 1 mM potassium chloride (KCl). Zeta potential measurements were taken on a Zeta Nanosizer (Malvern, United Kingdom).

### Differential Scanning Fluorimetry

Zaire GP (after the deletion of MLD and TM domain) at 10 μM, buffered by the addition of 10 μl PBS at pH 4.0, was mixed with 5 μl of SYPRO Orange dye (Sigma) at a 1:200 dilution, along with 10 μl of tolcapone or favipiravir (1, 10, and 40 μM) in 10% dimethyl sulfoxide (DMSO) or just 10% DMSO. The mixture was made up to a total volume of 25 μl. Samples were placed in a semi skirted 96-well PCR plate, sealed, and heated on an LightCycler 480 instrument (Roche) from 37°C to 95°C at a rate of 1°C ⋅min^−1^. Fluorescence changes were monitored with excitation and emission wavelengths at 465 and 580 nm, respectively. Reference wells, i.e., solutions without inhibitors, but with the same amount of DMSO, were used to compare the melting temperature (T_*m*_). Experiments were carried out in triplicate. The data were processed by LightCycler^®^ Thermal Shift Analysis.

### Cytotoxicity Assay

The cytotoxicity of tolcapone toward HeLa, A549, THP-1, and Huh-7 cells was evaluated using MTT assays (Tan et al., 2013). Briefly, approximately 90% confluent cells were plated in 96-well plates at 1 × 10^5^/ml, and the plates were incubated at 37°C overnight. Different concentrations of tolcapone were added, and the cells were incubated for an additional 48 h at 37°C. Then, the culture supernatant was discarded, and 100 μl of 0.5 mg/ml MTT solution was added to the cells. After incubating the cells for an additional 4 h at 37°C, the supernatant was removed, and the formazan crystals formed were dissolved in 150 μl of DMSO. The absorbance of the resulting solution at 570 or 450 nm was measured using an ELISA reader. The 50% cytotoxicity concentration (CC_50_) values were calculated.

### Computational Docking Analysis

Molecular docking calculations were conducted using the Vina protocol in Yinfo Cloud Platform^1^. The three-dimensional (3D) structure of compound tolcapone was constructed with energy minimization in MMFF94 force field. The crystal/NMR structure of PAP248–286 [RCSB Protein Data Bank (PDB) No. 2L3H] and Ebola GP [RCSB Protein Data Bank (PDB) No. 5JQ3] was downloaded from the RCSB Protein Data Bank^2^. The crystal ligand was separated and used to define the binding pocket. AutoDockVina (Trott and Olson, 2010) program was utilized to perform semi-flexible docking with maximum nine poses output after internal clustering.

### Statistics Analysis

Statistical analysis of the experimental data was performed using a one-way or two-way analysis of variance (ANOVA) test in GraphPad Prism 5.0 (San Diego, CA, United States); *p*-values < 0.05 was considered as statistically significant; the probability level is indicated by single or multiple asterisks (^∗^) (^∗^*p* < 0.05; **p < 0.01; ***p < 0.001). All results were expressed as means ± standard deviation (SD) of three independent experiments.

## Results

### Tolcapone Inhibits the Assembly of Semen-Derived Enhancer of Viral Infection Amyloid Fibrils

**FIGURE 1:**
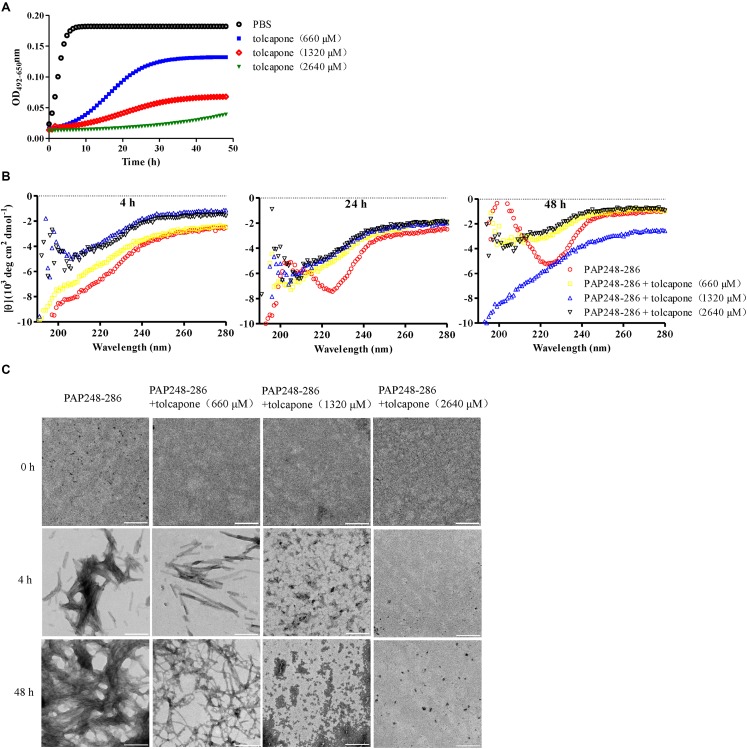
Tolcapone inhibits the assembly of semen-derived enhancer of viral infection (SEVI) amyloid fibrils. **(A)** PAP248–286 (220 μM) was incubated with tolcapone (660, 1320, 2640 μM), and the mixture was agitated at 1,400 rpm at 37°C. Fibril formation was detected by Congo red staining at the indicated time points (0, 2, 4, 8, 12, 24, 48 h). The Congo red staining measurements were plotted as a function of time and fitted by a sigmoidal curve using Origin data analysis software to determine lag time (*t*_*lag*_). **(B)** β-Sheet formation of SEVI fibrils was monitored by circular dichroism (CD) spectroscopy at the time point of 4, 24, and 48 h. SEVI was tested at 66 μM. The experiment was repeated once, and a similar result was obtained. **(C)** SEVI fibrils in the presence or absence of tolcapone at 0, 4, and 48 h were visualized by transmission electron microscopy (TEM). Scale bars indicate 200 nm.

Tolcapone was firstly assessed for its effects on inhibiting the spontaneous amyloidogenesis of PAP248–286, the well-characterized seminal amyloidogenic peptide. The aggregation kinetics of PAP248–286 alone or in the presence of tolcapone was analyzed by Congo red binding assay. Congo red is an amyloid-binding specific dye. It can specifically bind with amyloid fibrils, resulting in increased optical absorbance proportional to the amount of fibrils (Nilsson, 2004). The amyloid fibrils growing curve should indicate three phases, including a lag phase, a fast growth phase, and a steady equilibrium phase. For the pure PAP248–286, the lag phase was negligible (*t*_*lag*_ was around 0.06 h), and the growth phase remarkably turned to the equilibrium phase after 12 h (Figure 1A). However, co-incubation with different concentrations of tolcapone greatly increased the lag phase and lowered the maximum of optical absorbance compared to that of the PAP248–286 control (Figure 1A). The values of *t*_*lag*_ were 6.20 h (tolcapone at 660 μM), 10.86 h (tolcapone at 1,320 μM), and 44.15 h (tolcapone at 2,640 μM). The results demonstrate that tolcapone could effectively inhibit PAP248–286 aggregation.

The conformational conversion of PAP248–286 in the absence or presence of tolcapone was analyzed by far-UV CD spectroscopy. As shown in Figure 1B, the CD spectrum of PAP248–286 with or without tolcapone at the time point of 4 h (left panel) presented a major negative peak at around 200 nm, implying a mainly random coil structure (Sarr et al., 2018). At the time point of 24 h (middle panel) and 48 h (right panel), the CD spectrum of PAP248–286 presented a major positive peak at ∼202 nm and a minor negative peak at ∼222 nm, which is a typical spectrum of the β-sheet-rich conformation. However, for the PAP248–286 sample that was co-incubated with tolcapone, the CD spectrum was much the same as that at the 4 h, a mainly random coil structure. The results suggest that tolcapone inhibits the structural transition of PAP248–286 from random coil to cross β-sheet structure.

In order to further investigate the inhibitory effect of tolcapone on PAP248–286 aggregation, the morphological changes of PAP248–286 fibers in the presence or absence of tolcapone were observed using TEM. Robust, abundant, and mature fibrils formed by PAP248–286 were identified after agitation for 48 h. However, after co-incubation with tolcapone, the aggregation products were only short and thinner protofibrils and amorphous aggregates. When incubated with 2,640 μM tolcapone, limited amyloid fibrils were visualized even after agitation for 48 h (Figure 1C).

Besides PAP248–286, other amyloidogenic peptides derived from semenogelins (SEMs), including SEM1 86–107, have also been reported to enhance HIV-1 and EBOV infection (Roan et al., 2014; Bart et al., 2018). To investigate whether tolcapone also inhibits SEM1 86–107 aggregation, SEM1 86–107 was agitated in the presence of tolcapone, and the changes of absorbance of Congo red during incubation were monitored. Compared to PAP248–286, tolcapone could inhibit SEM1 86–107 fibrillogenesis at higher concentration compared to that for PAP248–286. Amyloid fibril formation of SEM1 86–107 could be partially inhibited by 15 times of tolcapone (Supplementary Figure S1). Taken together, the above results suggest that tolcapone effectively obstructed the fibrillogenesis of PAP248–286.

### Tolcapone Attenuates the Ability of Semen-Derived Enhancer of Viral Infection Fibrils to Enhance Pseudo-Ebola Virus Infection

**FIGURE 2:**
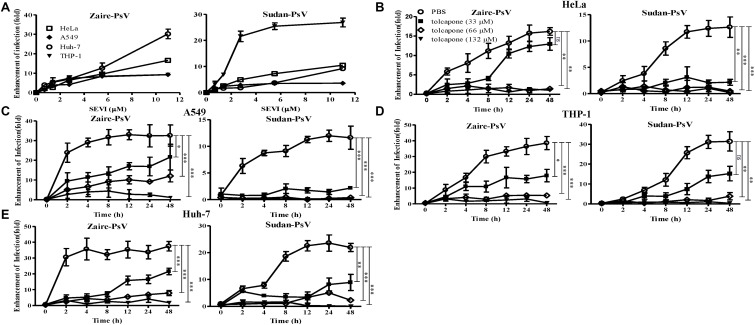
Tolcapone attenuates the ability of semen-derived enhancer of viral infection (SEVI) fibrils to enhance pseudo-EBOV infection. **(A)** Increasing concentrations of mature SEVI fibrils were mixed with Zaire-pseudovirus (PsV) (left panel) and Sudan-PsV (right panel) at room temperature for 10 min, respectively. The mixtures were used to infect HeLa, A549, Huh-7, and THP-1 cells. Luciferase activities were determined 72 h later, and results are reported as *n*-fold enhancement of infection relative to that without SEVI. The data are represented as the mean ± SD of three independent experiments. **(B–E)** In the presence of tolcapone, PAP248–286 lost the ability to enhance Zaire-PsV (left panel) and Sudan-PsV (right panel) infection in various cells after agitation. PAP248–286 was agitated with tolcapone at various concentrations as described above. Pellets were collected at the indicated time points by centrifugation, which were then mixed with Zaire-PsV (left panel) and Sudan-PsV (right panel). Mixtures were then used to infect HeLa **(B)**, A549 **(C),** THP-1 **(D),** and Huh-7 **(E)** cells. Luciferase activities were determined 72 h later. The final concentration of SEVI was 11 μM. The data represent the mean ± SD of three independent experiments. *p < 0.05; **p < 0.01; ***p < 0.001; one-way ANOVA.

It is reported that the fibrillar form of PAP248–286 aggregates, but not the monomer, possesses the ability to enhance EBOV infection (Bart et al., 2018). Therefore, we intended to determine whether enhancement of EBOV infection by PAP248–286 is lost in the presence of tolcapone, resulting from the inability of PAP248–286 to form fibrils. We first verified that mature SEVI fibrils could enhance EBOV infection in our EBOV/HIV-1_(NL4-3)Luc pseudoviral system, which is real mimicry of EBOV entry process and allowed to be used in a BSL-2 environment. The enhancement ability of SEVI on infection against Zaire PsV and Sudan PsV to various target cells during sexual transmission, including epithelium-derived HeLa and A549 cells, monocyte-derived THP1 cells, was examined. Human hepatocyte-derived Huh-7 cells, which represent the severely damaged target cell by EBOV infection, were also tested. Experiments were done with a low titer of virus to achieve optimal enhancing effect of SEVI. Our data demonstrated that SEVI enhanced both Zaire PsV (Figure 2A, left panel) and Sudan PsV (Figure 2A, right panel) infection of various cells lines. Next, we investigated whether tolcapone inhibited PAP248–286 fibril formation and, therefore, attenuated SEVI-induced enhancement of Ebola PsV infection. To eliminate the potential anti-EBOV activity of tolcapone itself, all the tolcapone-treated samples were centrifuged at 12,000 rpm for 5 min to remove the remaining free tolcapone. As expected, after agitating for 2–48 h, PAP248–286 effectively enhanced both Zaire PsV and Sudan PsV infection to various cell lines in a time-dependent manner, indicating an increase in fibril formation over time. In contrast, the PAP248–286 lost its ability to enhance pseudo-EBOV infection after agitation in the presence of various concentrations of tolcapone, and the effect was dose-dependent (Figures 2B–E).

SEVI has been well-documented to enhance HIV-1 infection. We also tested the antagonizing effects of tolcapone on SEVI-mediated HIV-1 infection. As shown in Supplementary Figure S2, tolcapone-treated PAP248–286 lost the ability to enhance both HIV-1 NL4-3 (X4-tropic) and HIV-1 SF162 (R5-tropic) infection even after agitation for 48 h. The results verify that tolcapone could effectively inhibit the conversion of PAP248–286 monomers to infection-promoting amyloids.

### Tolcapone Antagonizes the Assembly of Seminal Amyloid Fibrils

**FIGURE 3:**
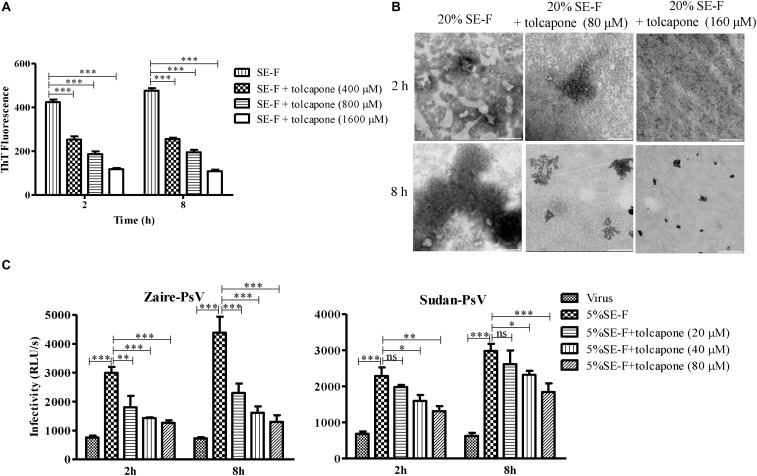
Tolcapone antagonizes the assembly of endogenous seminal amyloid fibrils. **(A)** Seminal amyloid fibril formation was antagonized by tolcapone in a dose-dependent manner. Fresh seminal fluid (SE-F) samples with tolcapone (0, 400, 800, and 1,600 μM) were agitated at 1,400 rpm at 37°C for 8 h. Fibril integrity at 2 and 8 h was detected by thioflavin T (ThT) assay. The data represent the mean ± SD of three independent experiments. ^∗^*p* < 0.05; ^∗∗^*p* < 0.01; ^∗∗∗^*p* < 0.001; two-way ANOVA. **(B)** Samples of seminal fibrils (1:5 dilutions) in the presence or absence of tolcapone (80 and 160 μM) after agitation for 2 and 8 h were visualized by transmission electron microscopy (TEM). Scale bars indicate 200 nm. **(C)** SE-F in the presence of tolcapone at the graded concentrations was agitated to allow fibril formation. The fibrils in the pellet were tested on their abilities to infect Zaire-PsV (left panel) and Sudan-PsV (right panel) in HeLa after 72 h. SE-F was finally diluted 20-fold. Shown are means ± SD of three independent experiments. ^∗^*p* < 0.05; ^∗∗^*p* < 0.01; ^∗∗∗^*p* < 0.001; two-way ANOVA.

The effect of tolcapone on the fibrillogenesis of fresh semen, in which endogenous amyloid fibrils have been removed by centrifugation, was detected *via* the enhancement of fluorescence intensity upon the binding of the commonly used amyloid-specific ThT dye (Nilsson, 2004). We found that SE-F alone displayed a slight increase in fluorescence intensity, suggesting the presence of newly formed seminal fibrils. Of note, tolcapone dose-dependently inhibited the new formation of seminal fibrils in SE-F (Figure 3A). We further confirmed the inhibitory effects using TEM assay. Consistent with the results of the ThT assay, fresh SE-F could form amyloid fibrils. However, in the presence of tolcapone, limited newly formed amyloid fibrils could be found in SE-F after 8-h agitation (Figure 3B). We further examined whether tolcapone inhibited the infection-enhancing properties of human SE-F. To eliminate the potential anti-EBOV activity of tolcapone itself, all the tolcapone-treated samples were centrifuged at 12,000 rpm for 5 min to remove the remaining free tolcapone. SE-F samples could enhance the infection by Zaire PsV and Sudan PsV to HeLa cells. However, incubated with tolcapone, SE-F gradually lost the ability to enhance Zaire PsV and Sudan PsV infection (Figure 3C). The results show that tolcapone could inhibit the newly formed seminal amyloid fibrils of human SE.

### Tolcapone Inhibits Oligomerization of PAP248–286 Monomers

**FIGURE 4:**
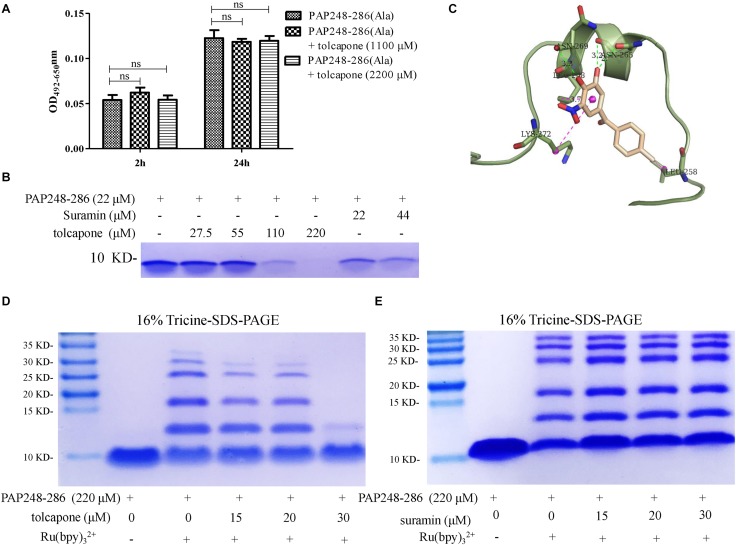
Tolcapone inhibits oligomerization of PAP248–286 monomers. **(A)** PAP248–286(Ala) (220 μM) were agitated with or without tolcapone. Fibril formation was detected by Congo red staining at 2 and 24 h. The data represent means ± SD of three independent experiments. **(B)** PAP248–286 at 22 μM was incubated with tolcapone or suramin at different concentrations at 37°C for 30 min. The remaining free PAP248–286 in the supernatants after centrifugation at 5,000 rpm for 10 min was recognized by Coomassic blue. **(C)** Presumed binding sites of tolcapone to PAP248–286. According to the computational docking results, tolcapone formed hydrogen bonds with ASN265 and ASN269, and it bound to LYS272, LEU258, and LEU268 by π–π interactions. **(D)** The effects of tolcapone on the oligomerization of PAP248–286 were assessed using 16% tricine-sodium dodecyl sulfate (SDS)-polyacrylamide gel electrophoresis (PAGE) and Coomassie blue staining. PAP248–286 monomer at 220 μM with or without cross-linking served as the control. **(E)** Suramin showed no ability to inhibit the oligomerization of PAP248–286.

Seminal amyloid fibrils are distinct from other amyloid fibrils due to its positive charges. We found that tolcapone failed to inhibit the fibril formation of PAP248–286(Ala), a mutant peptide in which the positively charged lysines and arginines were replaced with neutral alanines (Figure 4A). The results suggested that the tolcapone might target the positive charged residues in PAP248–286 to prevent PAP248–286 aggregation. We next investigated whether a potential interaction between tolcapone and PAP248–286 might account for tolcapone’s inhibitory activity on PAP248–286 aggregation. We found that after the co-incubation of tolcapone with PAP248–286, the level of PAP248–286 in the supernatant gradually decreased with increasing amounts of tolcapone, suggesting a binding of tolcapone with PAP248–286 (Figure 4B). Suramin, which has been shown to interact with PAP248–286 (Tan et al., 2019), was served as a positive control. We further applied mass spectrometry to examine the potential interaction between PAP248–286 and tolcapone. The parent ion of tolcapone has been detected at m/z 274.2 (Supplementary Figure S3A), and the parent ion of PAP248–286 was obtained at m/z 526.0 (Supplementary Figure S3B). When tolcapone was mixed with PAP248–286 at the molar ratio of 10:1, the parent ion for tolcapone alone or PAP248–286 alone could not be detected in the mass spectrometry of the mixture (Supplementary Figure S3C), which suggests a potential interaction between PAP248–286 and tolcapone.

In order to further validate the interaction between tolcapone and PAP248–286 at the atomic level, computational molecular docking analysis was conducted. The result showed that tolcapone binds to PAP248–286 and forms hydrogen bonds *via* ASN265 and ASN269 (Figure 4C). Besides, π–π interactions also existed in the PAP248–286–tolcapone complexes *via* LYS272, LEU258, and LEU268 (Figure 4C), forming strong electrostatic interactions. Of note, PAP248–253, PAP260–270, and PAP279–286 have been shown to be the amyloidogenic region with high fibril-forming propensity (Castellano and Shorter, 2012; Elias et al., 2014; Li et al., 2019). Tolcapone interacts with PAP248–286 by occupying the key residues in the amyloidogenic region of PAP248–286 and positive residue (Figures 4A,C), which might be responsible for the inhibitory effects of tolcapone on PAP248–286 aggregation.

The presence of low-n-order oligomers during the early stages of fibril assembly suggests a lag phase in fibril formation. Preventing the protein oligomerization has been shown a treatment option to inhibit amyloidogensis. We therefore used PICUPs to determine whether tolcapone prevents PAP248–286 monomer oligomerization. It was found that tolcapone significantly inhibited oligomerization of PAP248–286 dose-dependently. In the presence of cross-linking chemicals, the PAP248–286 monomer, which was used as a positive control, predominantly existed as a mixture of oligomers on the order of 2–10. The oligomerization of PAP248–286 (220 μM) did not proceed to completion in the presence of 30 μM of tolcapone (Figure 4D). As a negative control, suramin did not inhibit the early oligomerization of PAP248–286 (Figure 4E). The result suggests that tolcapone targeted the monomeric PAP248–286 and prevented its assembly into any sized oligomer.

### Tolcapone Modifies the Surface Charge of Semen-Derived Enhancer of Viral Infection and Blocks Amyloid-Induced Enhancement of Viral Infection

**FIGURE 5:**
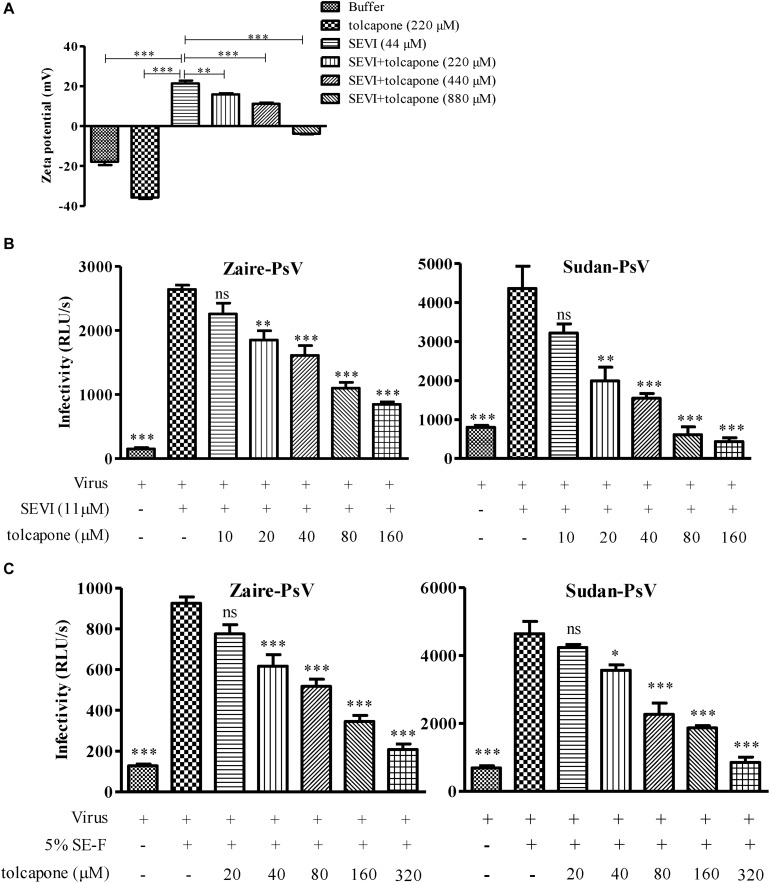
Tolcapone modifies the surface charge of semen-derived enhancer of viral infection (SEVI) and blocks amyloid-induced enhancement of viral infection. **(A)** Zeta potential of SEVI fibrils in the presence or absence of tolcapone. The results are the average values ± SD (*n* = 3). ^∗^*p* < 0.05; ^∗∗^*p* < 0.01; ^∗∗∗^*p* < 0.001; one-way ANOVA. SEVI **(B)** or agitated seminal fluid (SE-F) **(C)** was incubated with tolcapone at the indicated concentrations or phosphate buffered saline (PBS) for 15 min at 37°C. The mixtures were centrifuged, and the pellets were then incubated with Zaire-pseudovirus (PsV) (left panel) or Sudan-PsV (right panel), respectively. Infection of HeLa cells by measuring luciferase activity as described above 72 h post-infection. The data represent the mean ± SD of three independent experiments. ^∗^*p* < 0.05; ^∗∗^*p* < 0.01; ^∗∗∗^*p* < 0.001; one-way ANOVA.

The positive surface charge of SEVI fibril contributes to the facilitation of virion attachment to target cells and enhancement of viral infection. Thus, we investigated whether negatively charged tolcapone attenuates the interaction between SEVI fibrils and viruses. Zeta potential could be applied to determine the surface charge of a particle in solution. Tolcapone neutralized SEVI’s surface positive charge due to the fact that tolcapone significantly decreased the zeta potential of SEVI fibrils in a dose-dependent manner (Figure 5A). Tolcapone significantly influenced SEVI’s activity in enhancing infection of Zaire PsV and Sudan PsV by blocking the formation of virion–amyloid complexes. Mature SEVI fibrils were pretreated with tolcapone at 37°C for 15 min. Pellets were collected by centrifugation to remove the free tolcapone and then subjected to viral infection experiments. With increasing concentrations of tolcapone, mature SEVI fibrils gradually lost the ability to enhance Zaire PsV and Sudan PsV infection (Figure 5B). We next determined whether tolcapone inhibited the activity of endogenous mature fibrils in semen. The fresh SE-F was agitated at 37°C for 2 h to allow the newly formed amyloid fibrils. Then, the agitated SE-F was mixed with tolcapone at 37°C for 15 min. Mixtures were then centrifuged at 12,000 rpm for 5 min to collect the fibrils, which were used to determine their abilities to enhance Zaire PsV and Sudan PsV infection. It was shown that the newly formed amyloid fibrils in SE-F could greatly enhance both Zaire PsV (Figure 5C, left panel) and Sudan-PsV (Figure 5C, right panel) infection to HeLa cell. However, tolcapone significantly blocked endogenous seminal fibril-mediated enhancement of Zaire PsV (Figure 5C, left panel) and Sudan-PsV (Figure 5C, right panel) infection to HeLa cell.

### Tolcapone Antagonizes Semen-Derived Enhancer of Viral Infection-Induced Enhancement of Zaire Pseudovirus Binding and Internalization in HeLa Cells

**FIGURE 6:**
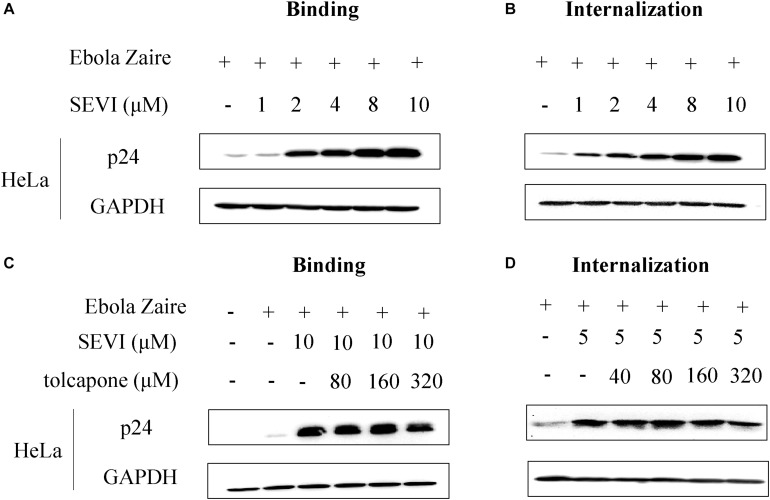
Tolcapone inhibits semen-derived enhancer of viral infection (SEVI)-induced enhancement of binding and internalization of Zaire-pseudovirus (PsV) to HeLa cells. Zaire-PsV was incubated with SEVI **(A,B)** or tolcapone-treated SEVI **(C,D)** then bound to HeLa cells on ice. Cells were either lysed in 1% Triton for 10 min on ice after 1 h (binding) **(A,C)** or warmed to 37°C for 1 h to allow viral internalization, washed with phosphate buffered saline (PBS), trypsinized, and lysed with 1% Triton (internalization) **(B,D)**. Lysates were used for determination of the level of p24 by Western blotting.

It is reported that SEVI-mediated enhancement of EBOV infection was involved in the promotion of viral binding and internalization (Bart et al., 2018). We first confirmed whether SEVI could enhance Zaire PsV binding and internalization in HeLa cells. Zaire PsV was pre-incubated with SEVI before binding to HeLa cells on ice for 1 h to test the binding ability or shifted to 37°C for an additional hour to permit internalization. Lysates were analyzed by Western blotting, and signal in the presence of SEVI was compared with Zaire PsV binding/internalization in the absence of SEVI. When incubated at 4°C, in which Ebola pseudoviruses could only bind to cells but not be internalized in cells because of deficiency of energy required for entry, SEVI could dose-dependently enhance Zaire PsV binding to HeLa cells (Figure 6A). Similarly, when incubated at 37°C after binding, the internalization of Zaire PsV in HeLa cells was dose-dependently increased (Figure 6B). The result confirmed that SEVI enhanced EBOV binding and internalization of HeLa cells.

Then, we determined whether tolcapone could antagonize SEVI-mediated enhancement of viral binding and internalization. Tolcapone was incubated with mature SEVI at 37°C for 15 min, and the tolcapone-bound fibrils were collected by centrifugation, which were then used to test their abilities to bind and internalize virus as described above. Tolcapone could dose-dependently decrease the enhancement of Zaire PsV binding (Figure 6C and Supplementary Figure S4) to HeLa cells and subsequent internalization (Figure 6D and Supplementary Figure S5) in HeLa cells.

### Tolcapone Is a Potent Entry Inhibitor of Both Zaire Pseudovirus and Sudan Pseudovirus

**FIGURE 7:**
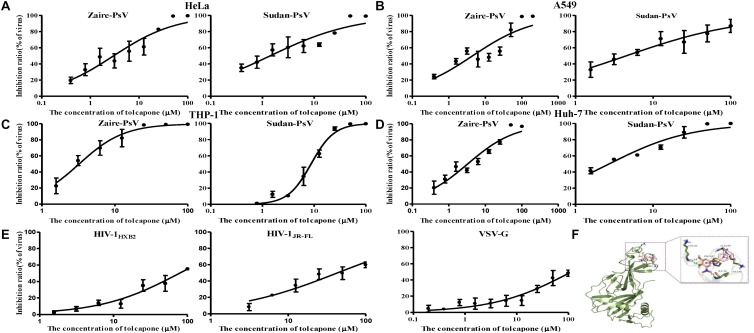
Tolcapone was a potent entry inhibitor of both Zaire PsV and Sudan PsV. The indicated concentrations of tolcapone were incubated with Zaire-PsV (left panel) or Sudan-PsV (right panel), respectively, for 30 min at room temperature. The mixtures were then added to prepared **(A)** HeLa, **(B)** A549, **(C)** THP-1, and **(D)** Huh-7 cells. Luciferase activities were measured at 72 h post-infection. Each sample was tested in triplicate, and the data are presented as the mean ± SD. **(E) %** Inhibition of HIV-1HXB2 (left panel), HIV-1JR-FL (middle panel), and VSV-G (right panel) infection by tolcapone. **(F)** Presumed binding sites of tolcapone to Ebola glycoprotein. According to the computational docking results, tolcapone formed hydrogen bonds with LYS56 and ILE38, and it bound to LYS190, ALA189, and PRO187 by hydrophobic interactions.

Interestingly, when we tested tolcapone alone with the pseudoviruses, we found that tolcapone had anti-EBOV entry activity. Pseudoparticles were exposed to tolcapone and then added to various cells. Infection rates were measured 72 h later by quantifying luciferase activity. The results showed that tolcapone efficiently blocked infection by both tested pseudoparticles in various cell lines. The half-maximal inhibitory concentrations (IC_50_) of tolcapone against the two pesudoviruses were similar and ranged between 1.41 and 8.76 μM for Sudan PsV and 2.99 and 4.33 μM for Zaire PsV (Figures 7A–D and Table 1). In contrast, a VSV-G pseudotyped virus expressing the VSV-G envelope and HIV-1 Env pseudotyped viruses, including HIV-1 HXB2 (X4 strain) and HIV-1 JR-FL (R5 strain), were used as negative control to evaluate the specificity of tolcapone for the EBOV envelope. Tolcapone could not effectively inhibit the infection against VSV-G pseudovirus and HIV-1 pseudoviruses at concentrations below 100 μM (Figure 7E), which suggested that tolcapone might be an EBOV entry inhibitor that targets EBOV envelope proteins specifically.

**TABLE 1:** Entry-inhibitory activity of tolcapone against Zaire PsV and Sudan PsV in various cell lines^a^.

**Cell lines**	**Zaire PsV Sudan PsV**
	**IC_50_ (μM) IC_50_ (μM)**
HeLa	2.99 ± 1.33 1.41 ± 0.74
A549	4.33 ± 2.26 4.49 ± 1.70
THP-1	3.24 ± 0.78 8.76 ± 1.23
HuH-7	3.29 ± 0.99 2.72 ± 0.61

*^*a*^Each sample was tested in triplicate, and the experiment was repeated three times. Data are presented as the mean ± SD.*

EBOV has a membrane envelope decorated by trimers of a GP (cleaved by furin to form GP1 and GP2 subunits) which is solely responsible for host cell attachment, endosomal entry, and membrane fusion. GP is thus a primary target for the development of antiviral drugs. Differential scanning fluorimetry (DSF) was performed to evaluate the potential interaction between tolcapone and EBOV GP trimer. DSF is a commonly used approach for detecting protein–ligand interactions. Upon binding to a folded protein, ligands stabilize the protein; thus, detecting an increase in the temperature at which the protein unfolds as a function of ligand concentration can serve as evidence of a direct interaction (Bai et al., 2019; Forneris et al., 2009). As shown in Supplementary Figure S6A, binding of 40 μM tolcapone to Zaire GP led to a shift of the T_*m*_ by 4.75°C. In contrast, the T_*m*_ of the mixture of favipiravir (RNA-dependent RNA polymerase inhibitor) and Zaire GP (after the deletion of MLD and TM domain) had no significant change (Supplementary Figure S6B).

To further explore the binding mode of tolcapone with Zaire GP, we used computational molecular docking to simulate the binding of tolcapone to EBOV GP and analyzed its possible binding sites. As shown in Figure 7F, tolcapone binds to EBOV GP, and they form hydrogen bonds *via* LYS56 and ILE38, and it bound to LYS190, ALA189, and PRO187 by hydrophobic interactions, which were consistent with the binding sites of inhibitors in related studies (Zhao et al., 2016).

### Tolcapone Exhibited a Synergistic Effect in Combination With Other Entry Inhibitor-Based Antivirals Against Zaire Pseudovirus Infection in Semen

Two FDA-approved drugs, sertraline (Zoloft) and bepridil (Vascor), have been shown to have both strong *in vitro* and *in vivo* anti-EBOV activity (Johansen et al., 2015). Sertraline is a selective serotonin reuptake inhibitor, and bepridil is a calcium channel blocker, both of which affect the fusion process late in the entry pathway. As shown in Table 2 and Supplementary Figure S7, combining tolcapone and bepridil in SE resulted in synergistic inhibitory activity against Zaire PsV infection in HeLa cells with CI values of 0.372 for 50% inhibition, including potency enhancement of 40.72-fold for bepridil and 2.88-fold for tolcapone. Combining tolcapone and sertraline resulted in strong synergistic inhibitory activity against Zaire PsV infection with CI values of 0.151 for 50% inhibition, including potency enhancement of 61.69-fold for sertraline and 7.4-fold for tolcapone. These results suggest that tolcapone and other entry inhibitors could be used in combination to enhance anti-EBOV activity.

**TABLE 2:** Combination index and dose reduction values for entry inhibition of Zaire-PsV infection by combining tolcapone with entry-inhibitory antivirals in semen.

**Drug combination, %Inhibitory Conc. (Molar ratio)**	**CI^a^**	**Tolcapone**			**Entry-inhibitory antivirals**		
		**IC_50_(nM)^b^**		**Dose reduction**	**IC_50_(nM)^b^**		**Dose reduction**
		**Alone Mixture**		**(Fold)**	**Alone Mixture**		**(Fold)**
**Tolcapone:Bepridil (133:1)**							
50	0.372	2637.62	915.67	2.88	279.74	6.87	40.72
90	0.216	29044.10	5619.65	5.17	1844.83	42.15	43.77
**Tolcapone:Sertraline (100:1)**							
50	0.151	2637.62	356.26	7.40	219.63	3.56	61.69
90	0.534	29044.10	13009.00	2.23	1514.23	130.09	11.64

*^*a*^CI, combination index. ^*b*^Data are the means of two independent experiments performed in triplicate.*

### Tolcapone Showed Low Cytotoxicity *in vitro*

**FIGURE 8:**
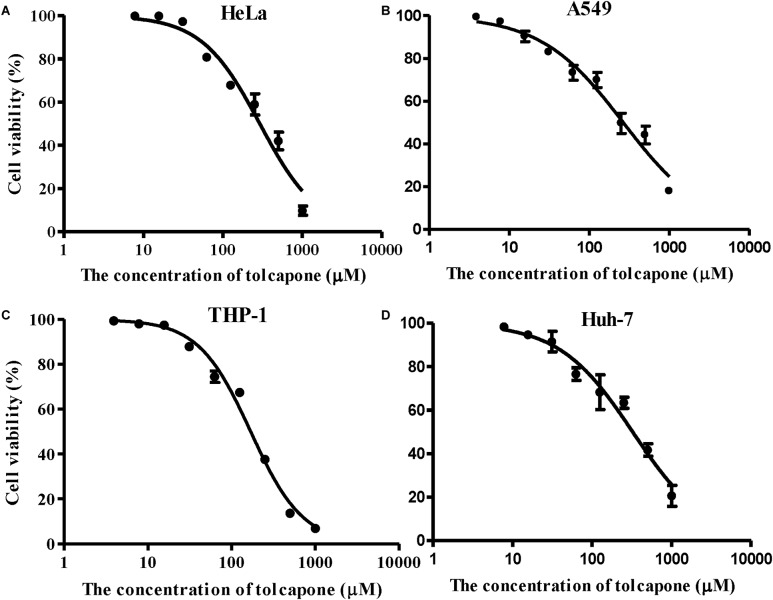
Cytotoxicity of tolcapone *in vitro*. **(A)** HeLa, **(B)** A549, **(C)** THP-1, and **(D)** Huh-7 cells. The concentrations of tolcapone were double diluted from 1,000 to 7.81 μM. Cytotoxicity was evaluated by 3-(4,5-dimethylthiazol-2-yl)-2,5-diphenyltetrazolium bromide (MTT) assay.

The potential cytotoxic effects of tolcapone on EBOV target cells (Huh-7 cell, THP-1, and A549 cells) and reproductive tract epithelial cells (HeLa cell) were evaluated using MTT assays. Tolcapone displayed low cytotoxicity *in vitro* toward all cells lines tested, with 50% cytotoxic concentration (CC_50_) values ranging from 169.3 to 324.8 μM (Figure 8 and Table 3). The selectivity index (SI = CC_50_/IC_50_) ranged from 52 to 98 (Table 3), suggesting that tolcapone might be safe for use *in vivo*.

**TABLE 3:** Cytotoxicity *in vitro* and selective index values of tolcapone against all tested cells^a^.

**Cell lines**	**Category**	**CC_50_ (μM)**	**IC_50_ (μM)**	**SI**
HeLa	Reproductive cancer cells	292.6 ± 41.6	2.99 ± 1.33	97.86
A549	Lung carcinoma cells	266.1 ± 34.3	4.33 ± 2.26	61.45
THP-1	Human macrophages	169.3 ± 14.5	3.24 ± 0.78	52.25
HuH-7	Hepatoma cells	324.8 ± 48.8	3.29 ± 0.99	98.72

*^*a*^The assay was performed in triplicate; Data are presented as the mean ± SD.*

## Discussion

Outbreaks of EVD are responsible for recent global health threats by causing many thousands of deaths and widespread disruption in the regions where the virus emerges. EBOV burden is directly associated with the rate of transmission. Although virus persisting in the semen has been well documented (Rodriguez et al., 1999; Bausch et al., 2007), little was known of its infectiousness. The role of sexual transmission of EBOV was recently confirmed in 2015 with the fact that at least one fatal case of EVD was contracted through sexual intercourse from a male survivor (Mate et al., 2015), and half of the Ebola outbreak flare-ups reported between 2015 and 2016 was likely associated with sexual transmission (Subissi et al., 2018). Recently, seminal amyloid fibrils, potential important host factors that facilitate EBOV sexual transmission, have been identified (Bart et al., 2018). Seminal amyloids greatly enhance EBOV infection and stabilize viral infectivity, thus, they are potential targets for intervention to prevent EBOV sexual transmission. Long-term persistence of infectious virus (Crozier, 2016; Diallo et al., 2016; Sow et al., 2016; Deen et al., 2017) and the infection-enhancing amyloids in semen underscore the critical need to develop rapid antiviral and anti-amyloid countermeasures to prevent virus transmission during unprotected sexual intercourse.

Although there has been great progress in developing anti-EBOV agents, to date, limited countermeasures were available to block EBOV sexual transmission. In this study, we found that tolcapone was a novel bifunctional topical agent counteracting sexual EBOV transmission both through targeting the infection-enhancing effects of amyloids in semen and by inhibiting virus entry. Tolcapone binds with amyloidogenic PAP248–286 and inhibits SEVI fibril formation (Figures 1, 4B), which results in the deficiency of PAP248–286 and fresh SE-F to enhance pseudo-EBOV infection (Figures 2, 3). Tolcapone also interacts with the mature amyloid fibrils and blocks fibril-mediated enhancement of pseudo-EBOV infection (Figure 5). More importantly, tolcapone antagonizes fibril-induced increase in binding and internalization of Zaire PsV in HeLa cells (Figures 6C,D). Tolcapone was found here to possess a dual mechanism of action on semen-derived amyloid fibrils and prevent the enhancement of EBOV sexual transmission by seminal fibrils.

Although tolcapone was found previously to have anti-EBOV activity by interfering with complex of the viral protein 35 (VP35) and nucleoprotein (NP), which is critical for viral RNA synthesis (Liu et al., 2017), we report here for the first time that tolcapone is also an effective EBOV entry inhibitor. Viral entry inhibitors are valuable as treatment options since blocking infection early in the life cycle will reduce cellular and tissue damage associated with the replication of incoming viruses. Tolcapone was shown to exhibit inhibitory activities against two pseudotyped viruses with values of IC_50_ ranging from 1.41 to 8.76 μM in various cell lines (Table 1 and Figure 7). Among several pseudotyped virions with different envelopes, tolcapone specifically inhibits pseudo-EBOV infection (Figure 7E). The GP of EBOV has been considered the only viral factor that mediates viral entry (Hunt et al., 2012). Our results showed that tolcapone binds with the GP trimer and inhibits viral entry (Supplementary Figure S6 and Figure 7F). Of note, tolcapone also exhibited synergistic effects with two small-molecule entry inhibitors of EBOV (Table 2) in semen, suggesting that tolcapone could be used alone or in combination to augment the antiviral potency and increase barriers against the development of drug resistance. Moreover, we evaluated the potential safety of tolcapone as a candidate microbicide. Our results showed that tolcapone displayed little cytotoxicity toward all tested cell lines *in vitro* (Table 3).

Several agents have been found in the past decade to minimize the viral infection-enhancing activity of semen amyloids in the context of stopping HIV-1 sexual transmission (Capule et al., 2012; Lump et al., 2015; Xun et al., 2015; LoRicco et al., 2016; Tan et al., 2019). However, most of them are at the stage of preclinical research. Tolcapone, shown in this study, is a repositioned compound with a newly identified activity as a potent SEVI fibril inhibitor and an EBOV entry inhibitor. It has been used in human and confirmed *in vivo* safety (Olanow and Watkins, 2007; Eggert et al., 2014). As a repositioned drug, tolcapone can bypass much of the early cost and time required to bring a drug to market. Compared to suramin, another repositioned compound that has been demonstrated to have anti-SEVI activity by us previously (Tan et al., 2019), tolcapone requires higher concentrations to inhibit PAP248–286 aggregation, which might potentially result from the fact that it possesses lower number and density of negatively charged residues. However, the abundant negative charge might imply non-specific binding in the human body and decrease the suitability for *in vivo* use. Moreover, our PICUP studies revealed that tolcapone inhibited PAP248–286 aggregation by the mechanism of disrupting PAP248–286 oligomerization at the early stage of fibrillogenesis, while suramin did not interfere with PAP248–286 oligomerization (Figures 4D,E). Suramin only coats on PAP248–286 and creates a steric barrier to block PAP248–286 interaction. It would therefore be expected that the most efficacious therapeutic agents would be those that block the early assembly processes associated with amyloid protein oligomerization.

Considering EVD outbreak and HIV-1, another important sexually transmitted pathogen, both prevalent in Africa, exploring bifunctional agent by targeting EBOV and seminal amyloids is of particular importance. The interplay between HIV and EBOV is not exactly known. However, the available literature verifies that EVD outbreak adversely affects the diagnosis and treatment of HIV/AIDS. Due to the interruption of routine health delivery services during the EBOV outbreak (Hira and Piot, 2016; Nagel et al., 2019), the chance of transmission and the number of prevalence and deaths caused by HIV/AIDS increased (Hira and Piot, 2016; Jacobs et al., 2017; Leno et al., 2018; Subissi et al., 2018; Nagel et al., 2019). Furthermore, HIV-positive EVD survivors have been documented and HIV-1 might play a role in supporting EBOV persistence in semen (Purpura et al., 2017). The situation of co-infection of HIV-1 and EBOV might be neglected due to the incomplete medical record. Seminal amyloids could enhance both HIV-1 and EBOV infection. Bifunctional agent by targeting EBOV and seminal amyloids might simultaneously inhibit EBOV and HIV-1 sexual transmission. Our results show that tolcapone could antagonize SEVI-mediated enhancement of EBOV and HIV-1 infection (Figure 2 and Supplementary Figure S2).

An important limitation of the present study is the inability to study the inhibitory effects with infectious EBOV. Because of its lethality, EBOV can only be handled in laboratories with biosecurity level-4 containment. Therefore, only few laboratories in the world can conduct EBOV research using the authentic virus (Dhama et al., 2018). Evaluation of the inhibitory effects of tolcapone on semen-mediated enhancement of infection against infectious EBOV strain is warranted.

## Conclusion

Exhibiting both excellent anti-amyloid and anti-EBOV effects, tolcapone might represent a prophylactic supplement or a lead product for the design of combination microbicide candidates to reduce the sexual transmission of EBOV.

## Data Availability Statement

All datasets generated for this study are included in the article/Supplementary Material.

## Ethics Statement

Semen (SE) samples were obtained from healthy lab members with written informed consent in accordance with the Declaration of Helsinki and the study was approved by the Human Ethics Committee of Southern Medical University, China. The patients/participants provided their written informed consent to participate in this study.

## Author Contributions

ST and SL conceived and designed the project. MQ, ZL, and YC performed the experiments and analyzed the data. JG and JP performed the mass spectrometric analysis. TQ and YQ collected semen samples. ZL and WX performed the DSF. LL and SL discussed the results and commented on the manuscript. ST and MQ wrote the manuscript.

## Conflict of Interest

The authors declare that the research was conducted in the absence of any commercial or financial relationships that could be construed as a potential conflict of interest.

## Footnotes

1 http://cloud.yinfotek.com/

2 http://www.rcsb.org/
