# Supplementary material for: Tolcapone Potently Inhibits Seminal Amyloid Fibrils Formation and Blocks Entry of Ebola Pseudoviruses
Source: Front Microbiol. 2020 Apr 30;11:504. doi: 10.3389/fmicb.2020.00504 (PMC7203225; doi:10.3389/fmicb.2020.00504)
Supplement: Supplementary file 1 [file Data_Sheet_1.pdf]

SUPPLEMENTARY INFORMATION

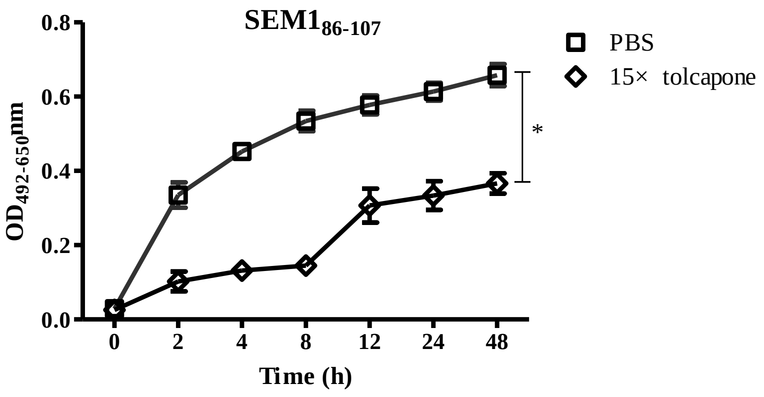

Figure S1. Tolcapone inhibits the fibril formation of SEM1 86-107. SEM1 86-107 (400 μM) were agitated with 15-fold excess of tolcapone. Fibril formation was monitored by Congo red staining at the indicated time points. The data represent the mean ± SD of three independent experiments. *$p<0.05$; one-way ANOVA.

A

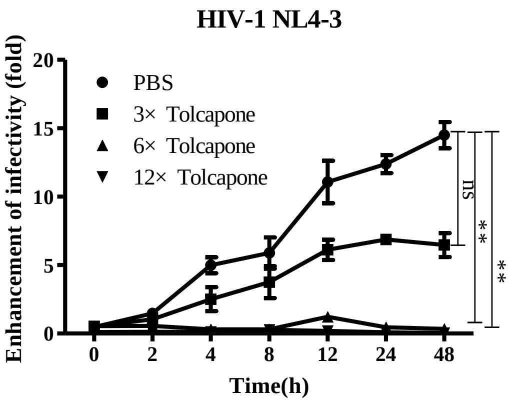

B

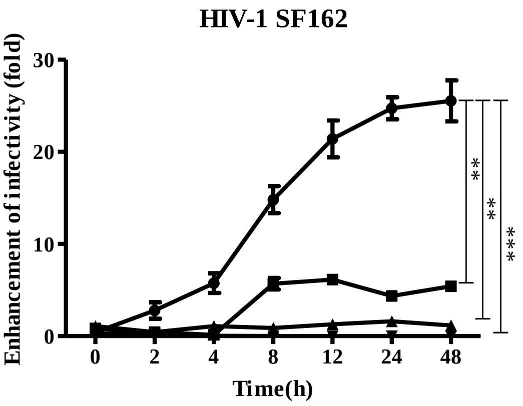

Figure S2. Tolcapone attenuates the ability of SEVI fibrils to enhance HIV-1 infection. The mixtures of PAP248-286 (11μM) and tolcapone at various concentrations were obtained at the indicated time points as described above. The samples were mixed with HIV-1 NL4-3 (left panel) and HIV-1 SF162 (right panel) and then added to TZM-b1 cells. Their abilities to enhance infection were shown by luciferase activities 72 h later. The data represent the mean ± SD (n=3). **$p<0.01$; ***$p<0.001$; one-way ANOVA.

A

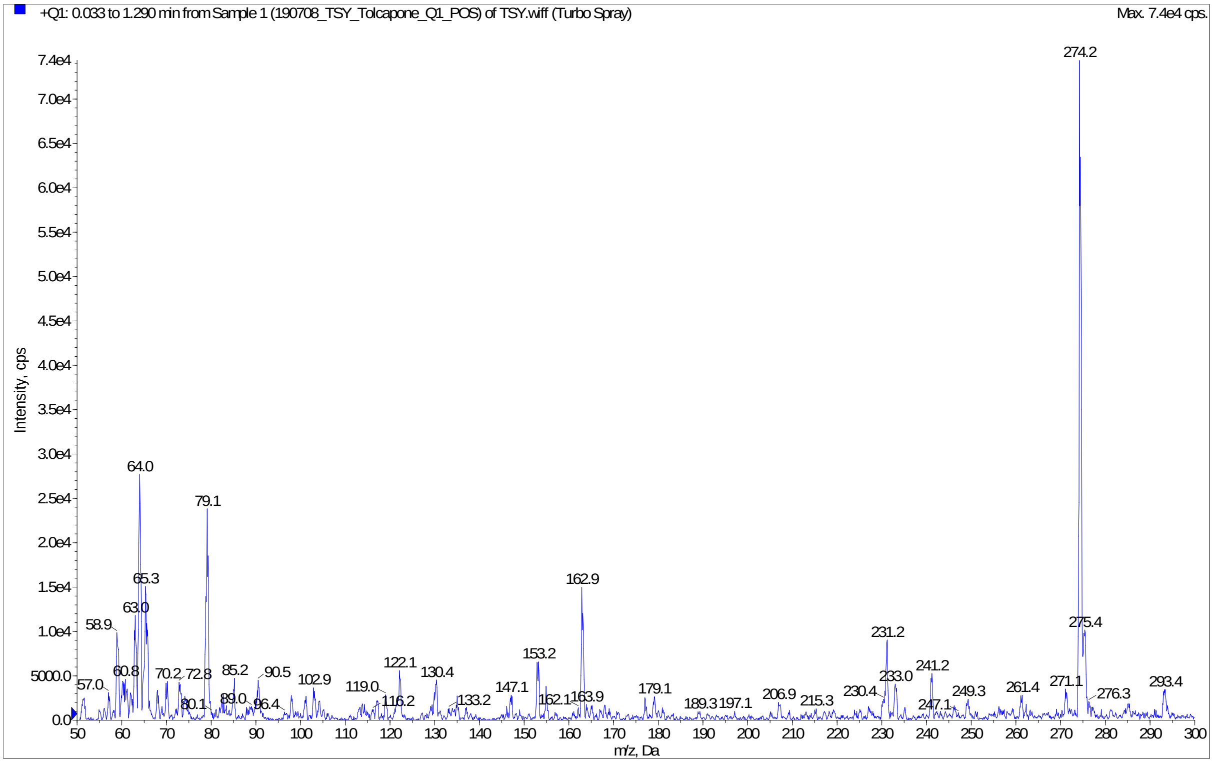
+Q1: 0.033 to 1.290 min from Sample 1 (190708_TSY_Tolcapone_Q1_POS) of TSY.wiff (Turbo Spray)
Max. 7.4e4 cps.
Intensity, cps
m/z, Da
274.2
64.0
79.1
162.9
65.3
63.0
58.9
153.2
231.2
275.4
122.1
241.2

B

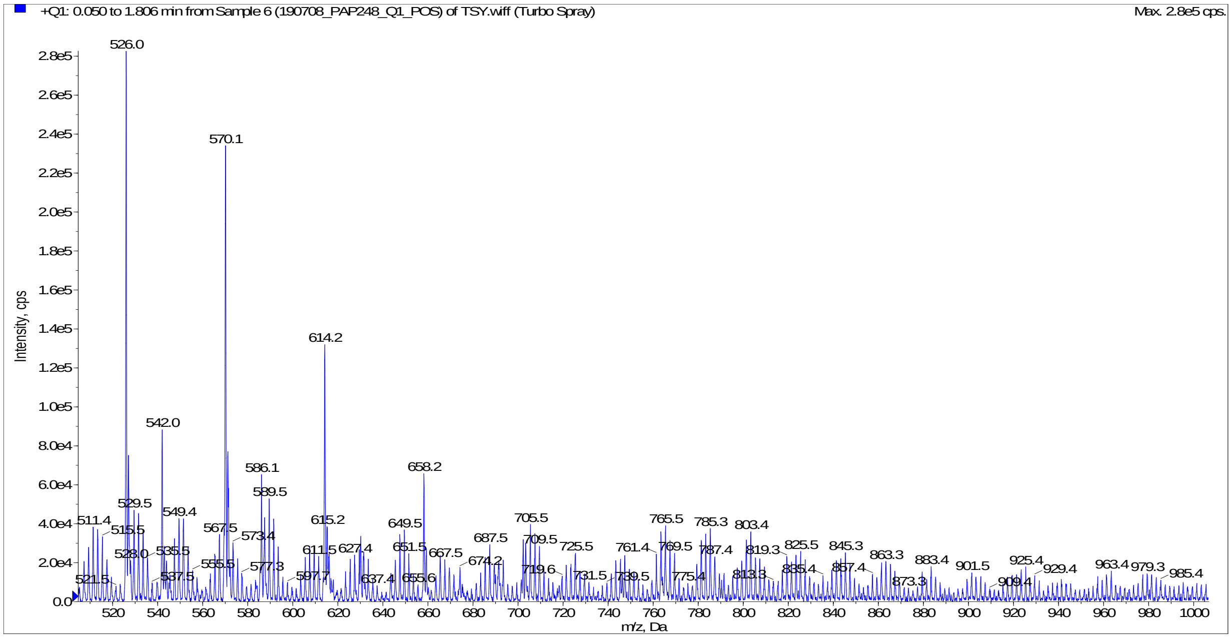
+Q1: 0.050 to 1.806 min from Sample 6 (190708_PAP248_Q1_POS) of TSY.wiff (Turbo Spray)
Max. 2.8e5 cps.
Intensity, cps
m/z, Da
526.0
570.1
614.2
542.0
586.1
658.2
589.5

C

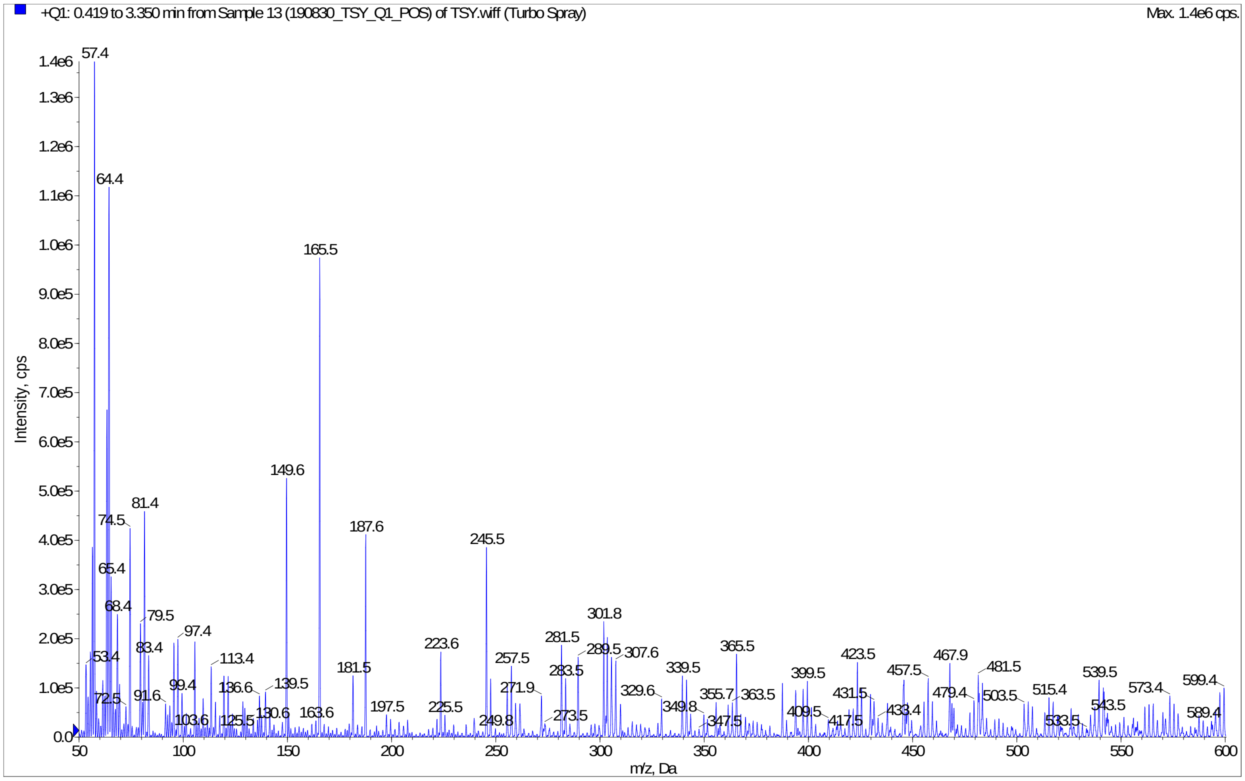

Figure S3. MS spectrum of tolcapone (A), PAP248-286 (B), and their complex at 1:10 (C). The changes of the corresponding parent ion indicate potential interaction between PAP248-286 and tolcapone.

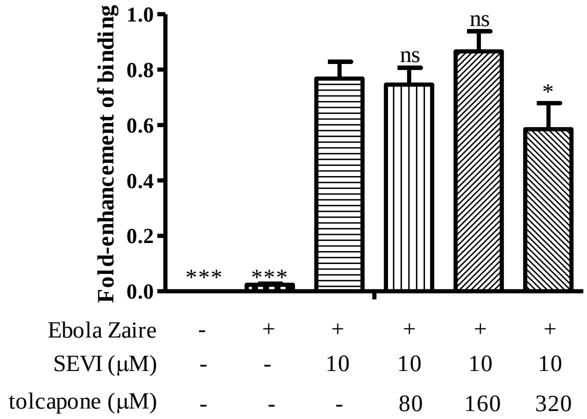

Figure S4. Relative quantification of p24 levels of Figure 6C by ImageJ analysis software. Results are representative of 3 independent experiments, *p<0.05; ***p<0.001; one-way ANOVA.

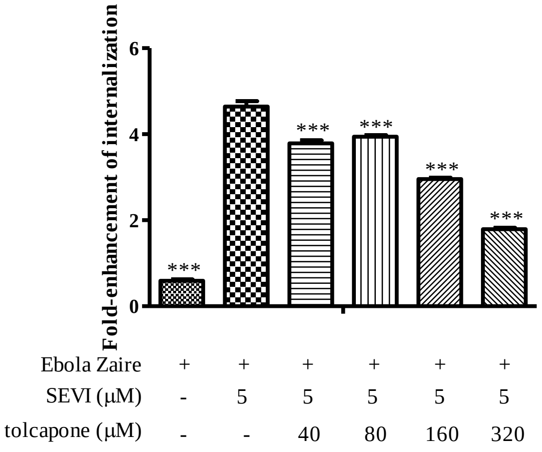

Figure S5. Relative quantification of p24 levels of Figure 6D by ImageJ analysis software. Results are representative of 3 independent experiments, ***p<0.001; one-way ANOVA.

A

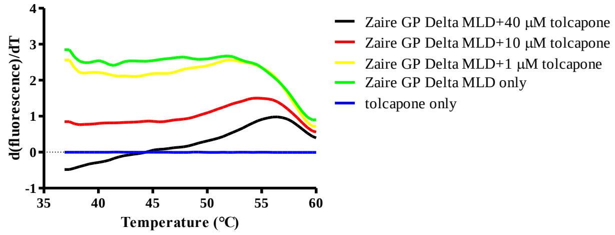

B

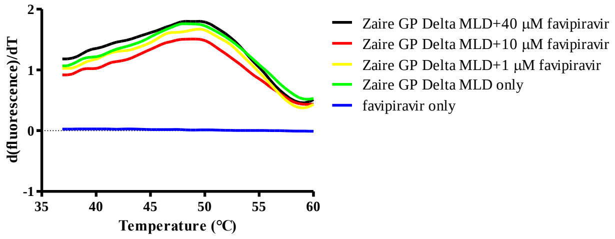

Figure S6 Melt curves of Zaire GP in the presence of tolcapone (A) or favipiravir (B) were demonstrated by DSF. 10 µM Zaire GP (after the deletion of MLD and TM domain) was mixed with 5 µL of SYPRO Orange dye (Sigma) at a 1:200 dilution, along with 10 µL of tolcapone or favipiravir (1 µM, 10 µM and 40 µM). Fluorescence changes were monitored with excitation and emission wavelengths at 465 and 580 nm respectively.

A

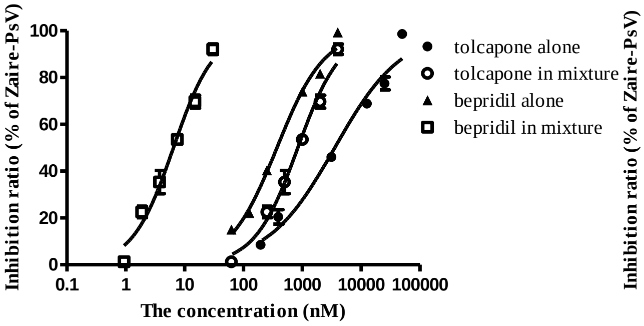

B

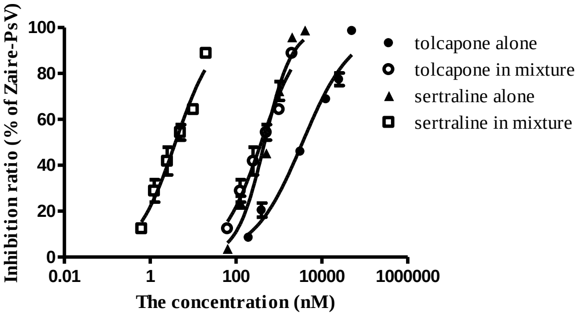

Figure S7. Synergism achieved by combining tolcapone with entry-inhibitory antivirals for inhibition of infection by Zaire-PsV in semen. Compounds were examined at fixed molar ratios individually and in combination. (A) tolcapone and bepridil (133:1); (B) tolcapone and sertraline (100:1). Luciferase activities were measured at 72 h post-infection in HeLa cells. Each sample was tested in triplicate, and the data are presented as the mean ± SD.
